# Personality traits and mental distress after COVID-19 testing. Prospective long-term analysis in a Viennese cohort

**DOI:** 10.3389/fpsyt.2023.1129794

**Published:** 2023-02-08

**Authors:** Claudia Guttmann-Ducke, Sonja Klinger, Rolf Ziesche, Bernd Otzelberger, Marco Idzko, Armin Ponocny, Simon Gabriel Prantl, Elisabeth Ponocny-Seliger

**Affiliations:** ^1^Division of Pulmonary Medicine, Department of Internal Medicine II, Medical University of Vienna, Vienna, Austria; ^2^Faculty of Psychology, Sigmund Freud Private University, Vienna, Austria; ^3^Faculty of Psychology, University of Vienna, Vienna, Austria

**Keywords:** anxiety, COVID-19 pandemic, depression, internal-external control, stress disorders - posttraumatic, stress psychological

## Abstract

**Background:**

Symptoms of mental stress are a hallmark of the COVID-19 pandemic. We hypothesized that just testing for COVID-19 could act as an effective stressor for persisting symptoms of mental distress including posttraumatic stress disorder. Our study aimed to determine whether personal beliefs on individual control and competence (locus of control, LoC) correlate with symptoms of mental distress and positive screening for post-traumatic stress disorder during a 9-month observational period.

**Methods:**

Between March and December 2021, we applied online versions of the Questionnaire on Competence and Control Expectations (FKK), the Depression, Anxiety, and Stress Score (DASS), the Short Screening Scale for DSM-IV Posttraumatic Stress Disorder (PTSD), and a medical history questionnaire for COVID-19 symptoms (visit 1). 48 hours after negative COVID-19 testing, DASS was repeated to address relief effects on mental distress (visit 2). Following 90 days (visit 3), development of mental distress was addressed by a combination of DASS and PTSD, while the possible long-term manifestation of PTSD was evaluated 9 months later (visit 4).

**Results:**

At visit 1, 7.4 percent of the total sample (*n* = 867) demonstrated a positive screening for PTSD, while after nine months (at visit 4), 8.9 percent of the remaining sample (*n* = 204) had positive screening results. The mean age was 36.2 years; 60.8% were women, 39.2% men. In contrast to individuals with negative PTSD screening, these participants demonstrated a significantly different LoC personality profile. This was confirmed by the results of both DASS and the COVID-19 medical history questionnaire.

**Conclusion:**

Following testing for COVID-19, individuals with positive long-term PTSD screening present with significantly different personality traits than those w/o suggesting that self-confidence and effective control over one’s own actions serve as a protective function against mental distress.

## Background

Rooted in individual experience and continuous adaptation in life, personal convictions about competence and control hold a vital impact on self-consciousness, self-assurance, and risk management (1). Owing to these deeply rooted personal feelings, individual beliefs can exert a lasting impact on behavior and communication within social groups. However, personal convictions and control strategies are challenged throughout periods of lasting oppressive stress (2, 3), such as the still ongoing COVID-19 pandemic. Systematic research on personal convictions commencing with the work of Rotter and his successors in the 1950s and 1960s (4, 5) introduced the perception of a “Locus of Control” [LoC; (6)]. It defines individual self-positioning based on two conditions: (a) the extent of a person’s control over its life and (b) the possibility to act effectively upon it. In line with this, LoC describes two general manifestations of personal conviction: individuals with high *internal* control capable of exerting close control over the majority of events in life, and those who believe in a predominantly *external* control rendering them susceptible to the control by others (7, 8). In line with this, a predominantly *external* LoC may predispose to repeated episodes of mental distress, anxiety, depression, or post-traumatic stress disorder (PTSD) (9, 10) precluding effective coping strategies. Thus, the effectiveness to cope with negative events depends on individual control flexibility. According to the *metacognitive* model of PTSD (11), this kind of flexibility is rooted in personal beliefs corresponding to a largely *internal* LoC. As a result, the development and, in particular, maintenance of long-term PTSD is likely to depend on self-consciousness and self-assurance.

The SARS-CoV-2 pandemic commencing in late 2019 and spreading throughout the world until now represents a prototypical example for a lasting and oppressive series of events challenging self-control, risk perception, communication, and social coherence (12). In line with this, numerous effects on behavior, development of mental distress, anxiety and depression have been described during this pandemic (13–15). Nonetheless, limited attention has been given to the possible influence of self-competence and control beliefs on self-assurance (16), and particularly on the possibility to develop symptoms of PTSD. Studies performed during the first SARS epidemic in 2003 and 2004 have reported that, for example, up to 10 percent of medical personnel developed PTSD (17). However, compared with the first SARS epidemic, both the time scale as well as the number of individuals affected during the current COVID-19 pandemic is by far more pronounced suggesting an even greater impact on psychological stability and well-being. Given the impact of LoC on control and coping strategies, we hypothesized that development and maintenance of symptoms characteristic for PTSD relate to individual beliefs about personal competence and control. Thus, in a Viennese cohort of 1,556 individuals, following *mandatory* COVID-19 testing with exclusively *negative* results, we conducted a prospective survey for symptoms suggestive of PTSD combined with an independent assessment of stress, anxiety, depression over a period of nine months.

### Hypothesis

Individual personality traits based on domains of competence and control beliefs (LoC) correlate with positive screening results for PTSD.

#### Additional questions

(1). What is the influence of domains of competence and control beliefs on mental health factors assessed using the standardized questionnaire ‘depression, anxiety and stress score’ (DASS)?
(2). Does a negative COVID-19 test result relate to development of anxiety, depression, and stress as assessed by DASS?
(3). Do people experiencing symptoms of COVID-19 and people who are subjectively symptom-free at visit 1, differ significantly concerning their DASS results?
(4). Is there a significant change in personal depression, anxiety, or stress experience between the three visits that DASS is applied?
(5). Do the results of PTSD screening at visit 1 differ significantly between clients subjectively experiencing COVID-19 symptoms and those who do not?

## Materials and methods

### Study design

We conducted a prospective questionnaire survey addressing the influence of personality traits on the development of stress, anxiety, depression, and posttraumatic stress disorder (PTSD) after COVID-19 testing. The study protocol was approved by the Ethics Committee of the Medical University of Vienna (EK1535/2020). The following validated inventories were used:

(a) Competency and Control Beliefs Questionnaire (FKK) (7);
(b) Depression Anxiety Stress Scales (DASS) (18); and
(c) German version of the Short Screening Scale for DSM-IV PTSD (19).

**FIGURE 1:**
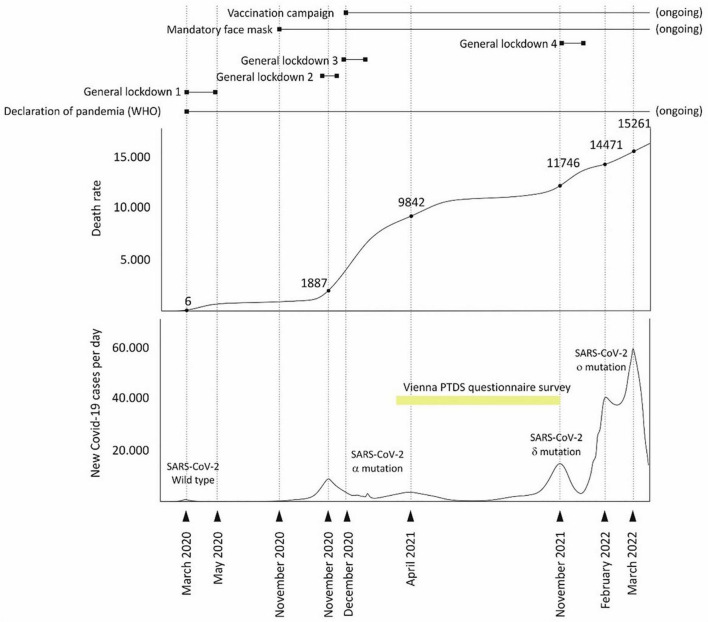
Timing of the Vienna post-traumatic stress disorder (PTSD) questionnaire survey during the COVID-19 pandemic in Austria.

At study begin, the third general lockdown had just ended and the rate of COVID-19-associated deaths in Austria was close to 10,000 (20). The permanent mask requirement had been in place since November; the vaccination campaign for medical staff and at-risk groups had started in December 2020. Randomization took place March 15-19, 2021, at the COVID-19 testing center, Wiener Stadthalle (Vienna; Figure 1). German-speaking subjects aged 18 years or more were recruited at the COVID-19 testing site.

After written informed consent, all test persons underwent pseudonymization and received a link via mail on their mobile phones. This approach provided the opportunity to complete the initial questionnaires during visit 1 at the test site while still waiting for the COVID-19 test results. Only fully completed inventories were evaluated; likewise, further participation in the survey was only possible if all inventories were completed. In case of missing answers, a reminder email was sent after 24 h.

At visit 1 (day 0), three validated tests (FKK, DASS, PTSD) and a medical history questionnaire addressing the major symptoms of COVID-19 infection (see Supplementary Table 2) were administered.
At visit 2 (two days after visit 1), DASS was applied. At visit 3 (90 days after visit 1), both DASS and the Short Screening Scale for PTSD were provided.

For the final investigation at visit 4 (270 days after visit 1), only the Short Screening Scale for PTSD was used.

### Survey instruments

Validated questionnaires:

Questionnaire on Competence and Control Beliefs (FKK) (7).

The FKK represents an enhancement of Rotter’s social learning theory developed by G. Krampen. The questionnaire records generalized expectations with regard to scope for action; these expectations and attitudes relate to personal experience, learned competence and the subjective evaluation of the given situation (7). It can therefore be expected that there is a connection between personal ideas of control and the development of PTSD and that this is generally true for anxiety, stress as well as depressive mood.

The seven scales of the FKK include *four* primary scales: (a) - (d); two secondary scales: (e) - (f); and one tertiary scale (g). These capture the following personality traits:

(a) Self-concept of one’s own abilities (FKK-SK) in the sense of recording possibilities for action against the background of self-confidence (Cronbach α = 0.72-0.82),
(b) Internality in the sense of having the power to determine one’s own life (FKK-I, Cronbach α = 0.65-0.76),
(c) Social externality or “powerful others control” as an expression of the dependence of an individual on the social environment (FKK-P, Cronbach α = 0.67-0.76), and
(d) Fatalistic externality or “chance control” (FKK-C) as a measure of the external determination of one’s own convictions (Cronbach α = 0.75-0.81).

The two secondary scales are calculated as follows:

(e) Self-efficacy = sum SK + I (FKK-SKI) and
(f) Externality = sum P + C (FKK-PC),

The tertiary scale is calculated as follows:

(g) Internality vs. externality (SK + I) - (P + C) (FKK-SKI-PC) serve as a criterion of self-efficacy without personal dependency vs. helplessness and external determination.

Depression-Anxiety and Stress-Score (DASS) (18), German version, which operationalizes the dimensions depression (α = 0.88), anxiety (α = 0.76), and stress (α = 0.86) with seven items each and is established in international research and clinical settings due to high user economy. The DASS shows high validity compared to the ADS and has higher sensitivity than the HADS (18). The DASS was used to screen for potential differential diagnoses of PTSD, i.e., depression, anxiety, and stress.

Short Screening Scale for PTSD (German version of the Short Screening Scale for DSM-IV posttraumatic-stress disorder). The scale includes nine validated items on thought and emotion avoidance consisting of: loss of interest, sense of alienation, numbness/deafness, unfulfilled plans for the future, sleep disturbances, distressing memories and distressing dreams/nightmares. Cut off value was ≥ 4. The procedure has a high internal consistency (α = 0.90), validity, and economy (19). Thus, it may be safely assumed that a possible PTSD development, even after negative COVID-19 testing, could be detected with sufficient accuracy (19). PTSD can only be diagnosed if symptoms persist for a minimum of 9 months, as described in the literature (21).

The test procedure allows for an additional self-categorization of mental traumata. In our study, five categorized traumata could be discerned: No trauma, anxiety, isolation, feeling of loss, and illness.

A categorized medical history questionnaire was used to collect the sociodemographic characteristics of the participants (see Supplementary Table 1) and individual symptoms of COVID-19 including concomitant and previously detected diseases.

### Data management and protection

All data were pseudonymized to avoid inference to individual persons; sensitive data are stored access-protected on the server of the MUW. ICFs are only accessible to authorized persons. After completion of the study, data were archived in the data management system according to legal requirements. Participants did not face any risk or individual benefit.

### Statistical analysis

Descriptive and inferential statistical analyses of the collected online data were performed using IBM SPSS 27 statistical software. The significance level was set at α = 5% and Bonferroni correction was applied to avoid alpha accumulation. Standardized effect size measures Cohen’s-d as well as η^2^ [partial eta-squared; (22)] and the relative risk according to odds ratio (OR) were used to interpret the content relevance of results.

In the context of descriptive statistics, mean (M) and standard deviation (SD), minimum (min), maximum (max) as well as median (Md) and interquartile range (IQR) were determined and quoted for characterization of metric parameters. The distributional assumption of the scores was tested and, in addition, the normal distribution of metric data can be assumed based on the validity of the central limit theorem for sample sizes n ≥ 30 (23, 24). Line plots with error indicators (± 1 SD) were created to illustrate the distribution of metric data. Absolute and relative frequencies and 95% confidence intervals, where appropriate, were calculated for categorical variables (gender, concomitant disorders, posttraumatic stress disorder).

Differences in the FKK profile with respect to PTSD (present vs. unremarkable) were tested using *t*-tests and Welch- test depending on the heterogeneity of variance (25). 95% confidence intervals were created for the probability of occurrence of PTSD at the survey time points. Assessment of distributional differences in two nominal scaled variables was based on cross-tabulations using chi-square tests (26). To examine change in depression, anxiety, and stress over time from three time points (day 0, 2, 90), multivariate mixed analysis of variance (mixed rmANOVA) was used to compare trends, when subjects tested positive for PTSD symptoms. Stepwise binary logistic regression was used on the FKK primary scales to predict a positive PTSD screening at visit 1 (27) and multiple linear regression models were finally used to examine the explanatory value of the FKK on the three DASS criteria. For feasibility, the premises of homoscedasticity, no multicollinearity, and normal distribution of the standardized residuals were tested for this purpose.

## Results

### Study population

**FIGURE 2:**
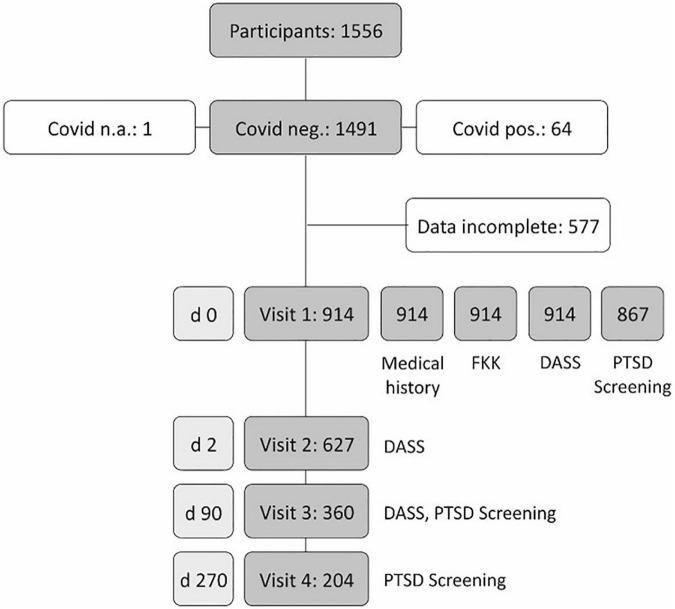
Case numbers and drop-out rates in the Vienna post-traumatic stress disorder (PTSD) survey.

Throughout a five-day period between March 15 and March 19, 2021, we recruited 1556 subjects (Figure 2). Sixty-four of these 1556 individuals (4.1%) were COVID-19 positive. For one participant, the test result could not be determined, leaving 1491 COVID-19-negative subjects to be included in the study. 577 survey protocols (38.7%) were incompletely processed resulting in 914 surveys for full analysis at visit one. Of these, 556 (60.8%) were women (Md 31 years, IQR 24-47), and 358 (39.2%) were men (Md 32 years, IQR 25-48). The median BMI was 23.04 (IQR 20.95-25.79) kg/(cm/100)^2^. Almost all test persons needed a negative test result to be able to pursue their professions; 56.9% were postgraduates, and 33.9% undergraduates. Almost half of the participants (48.2%) were employed as service professionals, 19.5%, and 12.3% as medical and technical professionals, respectively. 6.1% were retired, and in 13.9%, no information on occupation was available (Supplementary Table 1). At visit 1, 914 participants completed the medical history (for details, see Supplementary Table 2), as well as the FKK and DASS questionnaires. At visit 1, 867 participants completed the PTSD screening test. At visit 2, 627 completely processed PTSD test were received (72.3%), while at visits 3 and 4, 360 (41.5%) and 204 (23.5%), respectively were completed (see Figure 2).

### Subjectively perceived COVID-19 symptoms and mental state

Among the subjectively perceptible symptoms frequently reported during COVID-19 infection are symptoms of respiratory tract infection, such as fever, cough, and shortness of breath, as well as sore throat, head and muscle pain, eye pain, and diarrhea (see Supplementary Table 2). As a result, we classified the study participants dichotomously as symptom-free or symptomatic when at least one of these symptoms was reported. In total, 249 (27.3%) of 912 subjects with a complete protocol reported at least one of the symptoms. When testing for differences between these classified COVID-19 symptoms and the results of DASS at visit 1 using Welch tests, we found significant differences with higher scores for subjectively symptomatic participants with small effect sizes (Welch test: *p* ≤ 0.001; depressive states. d = 0.26, stress: d = 0.34, anxiety: d = 0.30). In line with this, testing the distributional difference of PTSD (yes vs. no) using cross-tabulation and chi-square testing showed a significantly higher rate of 11.1 percent for symptomatic vs. 6.0 percent for asymptomatic participants, *p* = 0.011; OR 1.95, 95% CI [1.16; 3.30].

### PTSD and DASS trajectories and self-reported assessment of competence and control (FKK) after COVID-19 test

**FIGURE 3:**
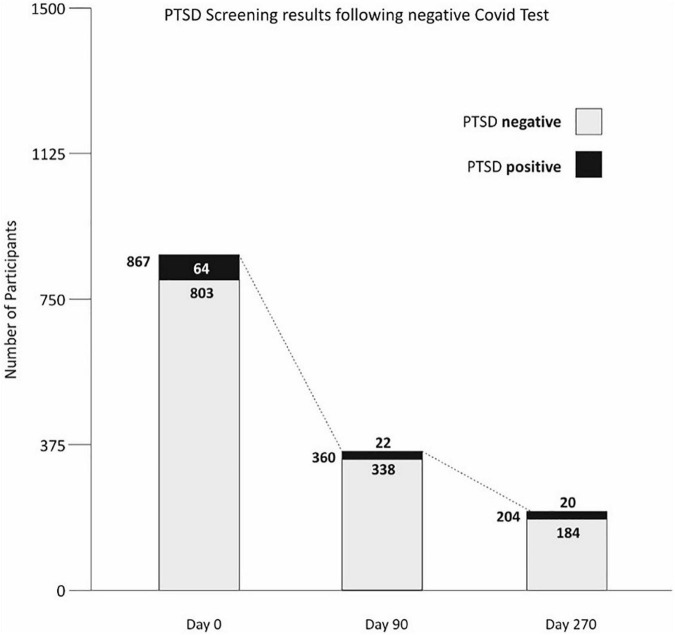
Post-traumatic stress disorder (PTSD) screening results during the survey.

Of the questionnaires returned at the start of study (*n* = 914), 47 (5.1%) PTSD questionnaires were not or only partially completed and thus not further processed. Of the remaining 867 PTSD questionnaires, 64 (7.4%; 95% CI [5.6%; 9.1%]) had a positive screening for PTSD (Figure 3). At day 90, 360 PTSD questionnaires were complete, with 22 subjects (6.1%; 95% CI [3.6%; 8.6%]) showing a positive PTSD screening. At day 270, we received 204 complete PTSD questionnaires, with 20 (9.8%; 95% CI [5.7%; 13.9%]) showing a positive screening for PTSD. When testing the stability of PTSD symptoms over time, five (2.4%) of the 204 participants showed a positive PTSD screening throughout the entire survey period. For a further 22 (10.8%) of the test persons, an inconsistent PTSD pattern was recorded. As a result, a total of 27 individuals (13.2%), PTSD was registered at least at one time point during the 9-month survey Figure 3.

The change in depression, anxiety and stress was tested on the basis of the DASS surveys (18) on day 0, after 2 days, and after 90 days, taking into account the PTSD status (cut off ≥ 4) by means of two-factor (3 × 2) mixed ANOVA. The limited sphericity using the ε-factor according to Huynh-Feldt had to be taken into account as a test requirement. The analyses were performed using the complete protocols with n = 354 (no PTSD) and n = 23 (PTSD) per protocol. The interaction of PTSD groups x time showed significant results (p’s < 0.05) for all three symptoms with small effects (η^2^ ≥ 0.01), so that the two main effects had to be interpreted differentially *post hoc*. The results for all three scales of the DASS showed significantly higher scores for PTSD (p’s < 0.001), each with significant effects for depression (η^2^ = 0.26), anxiety (η^2^ = 0.26) and stress (η^2^ = 0.19). The increase in depression scores for those with PTSD over 90 days was steady but not significant (*p* = 0.252, η^2^ = 0.06). Similarly, a non-significant increase was observed for anxiety (*p* = 0.166, η^2^ = 0.08) and stress (*p* = 0.532, η^2^ = 0.03).

**FIGURE 4:**
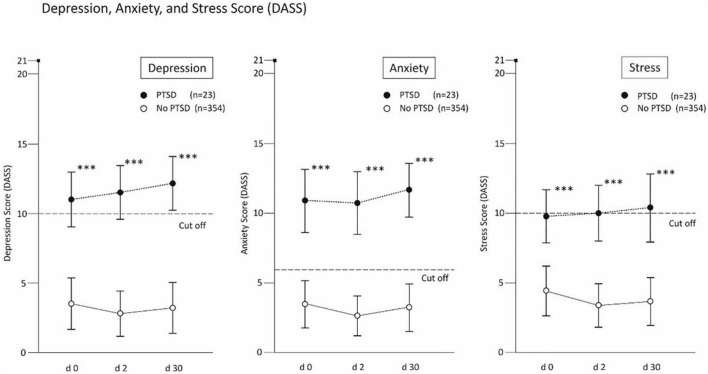
Results of the depression, anxiety and stress score in cases w/o post-traumatic stress disorder (PTSD). ^**^p < 0.01 and ^***^p < 0.001.

The trajectories of depressive states, anxiety and stress for participants with and without PTSD symptoms are illustrated in Figure 4.

**FIGURE 5:**
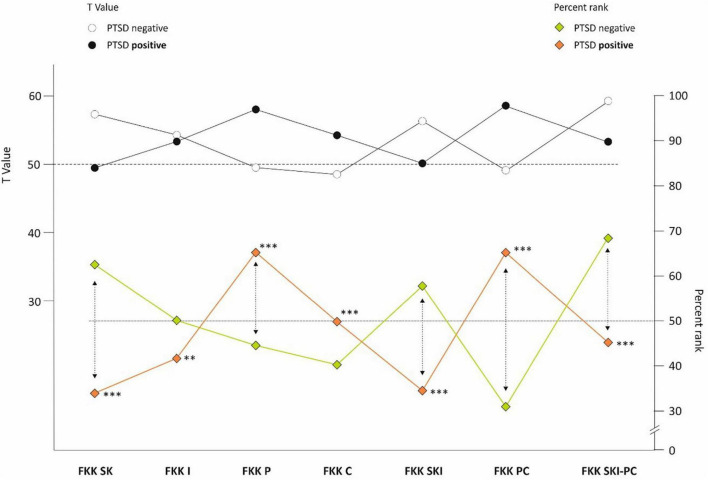
FKK profile in cases w/o post-traumatic stress disorder (PTSD). ^**^p < 0.01 and ^***^p < 0.001.

When comparing the personality profiles according to FKK with the PTSD scores at baseline, a significantly different personality profile was observed between individuals with (n = 62) and without positive PTSD screening (*n* = 782). Figure 5 (upper panel) demonstrates the FKK results as T-scores for all scales of FKK (μ = 50, σ = 10). Using t-test with Bonferroni adjustment (α* = 0.0071), the differences between the two PTSD groups was found significant for all scales (*p* ≤ 0.007) including the differences between PTSD groups with small to moderate effect sizes (*d* between 0.36 (for FFK-I) and 0.79 (for FKK-SKI-PC).

### Predictive power of LoC

On the basis of *n* = 844 protocols for visit 1 (day 0), the predictability of the criterion occurrence of PTSD was tested using binary logistic regression on the basis of the 4 FKK primary scales SK, I, P, C. The model fit was assumed using the non-significant Hosmer-Lemeshow test, *p* = 0.466. The model fit could be assumed based on the non-significant Hosmer-Lemeshow test, *p* = 0.461. By means of stepwise backward selection, FKK-SK (*p* = 0.001, OR = 0.92, 95%-KI [0.87; 0.97]) remained as a protective factor and FFK-C (*p* = 0.009, OR = 1.08, 95%-KI [1.02; 1.36]) as a risk factor with significant explanatory value for the occurrence of PTSD in the last model step. The coefficient of determination for the explained proportion of variance according to Nagelkerke’s R^2^ reached 10.4%.

Similarly, in order to assess the explanatory value of the four primary FKK domains FKK-SK, FKK-I, FKK-P, FKK-C for the three DASS criteria, multiple linear regressions were performed based on 872 cases. By means of stepwise backward selection, the predictors for the prognosis of depressive states, stress and anxiety were used accordingly. The results suggest that FKK-I should be excluded as a non-significant predictor for prognosis of depressive states, stress, and anxiety. For prediction of depressive states, the predictors FKK-SK (β = −0.30), FKK-P (β = 0.21) and FKK-C (β = 0.14) have small to moderate effect sizes. The same is true for prediction of stress using FKK-SK (β = −0.26), FKK-P (β = 0.22) and FKK-C (β = 0.09) as predictors. For anxiety, FKK-SK (β = −0.32), FKK-P (β = 0.22) and FKK-C (β = 0.10) each have a comparable predictive power (p’s < 0.01). The explained variance ratio R^2^_*adj.*_ reached 30.5% for depressive states, 23.8% for stress and 28.6% for anxiety.

## Discussion

The present long-term study took place from mid-March 2021 until December 2021 at the Wiener Stadthalle, the largest COVID-19 test site in Vienna, shortly after the advent of the α-variant of the SARS CoV-2 virus in Austria. This was a time characterized by widespread media coverage of rising morbidity and mortality rates in Austria. Since November 2020, it had been mandatory to wear FFP2 masks at any location outside the immediate living areas. Frequent, even daily, testing for COVID-19 by nasal swabs was obligatory to participate in professional and social life. Presuming that (a) COVID-19 testing, independent of its outcome, might act as a trigger for psychological stress, and (b) following the hypothesis that individual beliefs about self-competence and action control would significantly contribute to this kind of negative stress, randomly chosen individuals with negative COVID-19 test results were invited to participate in the study.

Locus of Control (LoC) is considered central to individual personality and as such, part of the theory of metacognition (28–30). Ongoing research on LoC has confirmed the influence of personality traits on different fields of psychology, such as health psychology, clinical psychology, and differential psychology (31, 32). It represents a dualistic concept of self-perception, self-control, and self-efficacy ranging from a predominantly internal LoC capable of exerting effective self-assessment and control to an external LoC largely depending on the beliefs and actions of others (33). Given these effects, it is feasible that the long-term restrictions imposed by the COVID-19 pandemic can trigger psychological effects based on the manifestations of LoC. In line with this, it has already been demonstrated that personality traits could pose a significant risk for COVID-19-associated mental stress (12, 34).

The study addressed the questions whether personality traits according to Rotter’s Locus of Control (LoC) would correlate with (a) symptoms indicative of stress, anxiety, and depression, and (b) with a positive screening for post-traumatic stress disorder (PTSD). To this end, we applied the advanced questionnaire on competence and control beliefs (FKK) by Krampen et al. (7, 33).

The FKK profiles [(7); primary scales: SK, I, P, C; secondary scales: SKI, PC; tertiary scale: SKI – PC] observed in participants with positive screening results for PTSD indicate a low ability self-concept, low internality, high social externality, and high fatalistic externality in this group. The test’s primary scales demonstrate the missing alternatives for action, low self-confidence, and self-awareness (SK), the poor representation of personal interests, efficacy of action and success rates (I), the extreme dependency on powerful others combined with personal helplessness as well as an overwhelming acceptance of external control (P) with low rationality and an intense belief in fate (C). The secondary scales of FKK emphasize both passivity, insecurity in action and low self-confidence (SKI), as well as socially conformist behavior combined with high helplessness, dependency from others and intense fatalism (PC), while the tertiary scale (internality vs. externality) in persons with positive PTSD screening stresses their extreme external LoC, their exceptionally low autonomy, passivity, and dependency on chance. This is in line with previous results demonstrating that low control beliefs and insufficient coping strategies related to it have a high impact on both development and maintenance of PTSD (35). This concept was confirmed by model tests in our setting, where the FKK-SK scale (*p* = 0.001, OR = 0.92, 95%-KI [0.87; 0.97]) can be regarded as a protective factor and the FFK-C scale (*p* = 0.009, OR = 1.08, 95%-KI [1.02; 1.36]) as a risk factor for the criterion occurrence of PTSD. The results of our study indicate that personality traits favoring low self-confidence and high externality could act as a precondition for the development of a post-traumatic stress disorder in the COVID-19 pandemic. This state of mind has already been encountered during the pandemic (36), reflecting a situation characterized by the continuous media presence of infection rates, mortality rates and challenges to access the health system (37), likely evoke feelings of utter helplessness (38).

The fact that participants with low internality scores and positive screening results for PTSD were also significantly more likely to report symptoms of a COVID-19 infection, without being ill may demonstrate the metacognitive power of self and external perception (11). This notion is further evidenced by the significantly varying scores for depression, anxiety, and stress (DASS) between individuals with and without positive PTSD screening (Figure 4; *p* < 0.001). Given the tendency of PTSD to stabilize over longer time intervals, it may be noteworthy that all DASS scores increase in PTSD screening positive individuals, in particular the scores for depression and anxiety, albeit not significantly (Figure 4). This corresponds with the observation that governmental action and media coverage is capable of generally increasing feelings of anxiety, depression, and stress (35, 36).

In this study, 14 men (3.9%) and 50 women (9%) of the total sample described PTSD symptomatology at study entry. Trauma defines that the affected person experiences a threatening situation, which is assessed as vital threatening and is accompanied by the feeling of helplessness and being at the mercy of the situation, as well as the shaking of the self and world view. Statistically, two-thirds of the world’s population experience trauma in their lifetime, with one-third developing PTSD and two-thirds coping with trauma through their own experiential and competence mechanisms and social systems (39). Traumatic experiences related to the COVID-19 pandemic were described by 43.2% of women and 32.2% of men with fear and isolation factors having the highest response. Psychological distress from the COVID-19 pandemic is shown in a nationwide study that generalized anxiety, depression, and distress increased significantly over the course of the pandemic (36). In this study individuals with positive PTSD screening results had significantly higher scores (p > 0.001) at all three time points in the three scales of depression, anxiety, and stress, but no significant differences for depression, anxiety, and stress. PTSD screening negative subjects had no depression, anxiety, and stress symptoms during the nine-month study period. Furthermore, the results suggest that the predictors of the primary scales FKK-SK, -P, and -C of the locus of control are suitable for predicting depressive states, anxiety, and stress with small to moderate β-weightings for prediction, respectively, *p* < 0.01.

## Limitations

The main weakness of this study is our lack of knowledge if psychological or psychiatric help in any form had been used by the participants. Furthermore, the cut-off scoring procedure in the DSM IV screening test for PTSD may not correspond to the latest DSM V convention. In addition, it was not possible to assess the influence of COVID-19 coverage in the media on the mental state of the participants.

## Data availability statement

The original contributions presented in this study are included in this article/Supplementary material, further inquiries can be directed to the corresponding author.

## Ethics statement

The studies involving human participants were reviewed and approved by Ethics Committee of the Medical University of Vienna, Approval No. 1535/2020. The patients/participants provided their written informed consent to participate in this study.

## Author contributions

All authors listed have made a substantial, direct, and intellectual contribution to the work, and approved it for publication.
