# Supplementary material for: Personality traits and mental distress after COVID-19 testing. Prospective long-term analysis in a Viennese cohort
Source: Front Psychiatry. 2023 Feb 8;14:1129794. doi: 10.3389/fpsyt.2023.1129794 (PMC9944018; doi:10.3389/fpsyt.2023.1129794)
Supplement: Supplementary file 1 [file Table_1.pdf]

Supplement 1

Table 1.

**Demographic characteristics of participants**			
**Total**			
		914	
**Gender**			
Women		556	60,8%
Men		358	39,2%
**Age**			
	Mean	36,2	
	min	18	
	max	80	
**BMI**			
Women		23,5	
Men		24,6	
**Occupation**			
Service profession		441	48,2%
Medical profession		178	19,5%
Technical profession		112	12,3%
Retired		56	6,1%
No specification		127	13,9%
**Education**			
Undergraduate		310	33,9%
Postgraduate		520	56,9%
Lower		61	6,7%
No specification		23	2,5%
