# Supplementary material for: Personality traits and mental distress after COVID-19 testing. Prospective long-term analysis in a Viennese cohort
Source: Front Psychiatry. 2023 Feb 8;14:1129794. doi: 10.3389/fpsyt.2023.1129794 (PMC9944018; doi:10.3389/fpsyt.2023.1129794)
Supplement: Supplementary file 2 [file Table_2.pdf]

Supplement 2

Table 2.

**Self-categorized symptom characteristics of participants**		
**Total**		
	914	100%
**Respiratory infection**		
Yes	24	2.6%
No	890	97.4%
**Cough**		
Yes	54	5.9%
No	860	94.1%
**Shortness of breath**		
Yes	31	3.4%
No	883	96.6%
**Sore throat**		
Yes	78	8.5%
No	836	91.5%
**Eye pain**		
Yes	5	0.5%
No	909	99.5%
**Joint pain**		
Yes	50	5.5%
No	864	94.5%
**Diarrhea**		
Yes	55	6.0%
No	859	94.0%
**Fever > 37.5°C**		
Yes	27	3.0%
No	887	97.0%
